# Acute mesenteric venous thrombosis in a pregnant woman at 35 weeks of gestation: a case report and review of the literature

**DOI:** 10.1186/s12884-018-2126-1

**Published:** 2018-12-11

**Authors:** Xiuting Guan, Lina Huang, Liping Li

**Affiliations:** Department of Obstetrics and Gynecology, Guangzhou First People’s Hospital, School of Medicine, South China University of Technology, Guangzhou, China

**Keywords:** Mesenteric venous thrombosis, Pregnancy, Acute abdomen, Intestinal ischemia

## Abstract

**Background:**

Mesenteric venous thrombosis (MVT) is an infrequent thrombotic event that can cause devastating intestinal hemorrhagic ischemia. The mortality rate among patients with acute MVT ranges from 20 to 50%. Occurrence of MVT in pregnancy is quite rare. In this case report, we describe a pregnant woman who presented with acute MVT at 35 weeks of gestation.

**Case presentation:**

Our case was a 26-year-old primigravid woman at 35 weeks gestation. She presented to Guangzhou First People’s Hospital with complaints of abdominal pain, nausea and vomiting. The second day after admission, she complained of more intense abdominal pain, anorexia, vomiting and abdominal distention that were out of proportion to physical signs. An emergency exploratory laparotomy was performed. The entire ileum, part of the jejunum and part of the ascending colon were gangrenous, and thromboembolism was discovered in the corresponding mesenteric veins. The necrotic intestine was resected and an end-to-end jejunum-colon anastomosis was performed. A cesarean section was performed to remove the placenta and fetus, which had expired. Histopathological analysis revealed extensive edema, hemorrhage, inflammatory infiltration and necrosis in the resected bowel, and widespread thrombosis in mesenteric venous lumens.

**Conclusion:**

The diagnosis of MVT during pregnancy is very difficult due to its low incidence, and non-characteristic symptoms, signs and laboratory results. MVT may be the underlying cause of severe abdominal pain during pregnancy and should be included in the differential diagnosis of pregnant patients with an acute abdomen.

## Background

Mesenteric venous thrombosis (MVT) is an infrequent thrombosis with an estimated incidence of only 2.7 per 100,000 person-years [1]. MVT can cause devastating intestinal hemorrhagic ischemia, peritonitis, sepsis and shock [2]. The mortality rate among patients with acute MVT ranges from 20 to 50% [2]. A wide range of prothrombotic states have been linked to MVT, including cancer, trauma, intraabdominal inflammatory conditions, the postoperative state, oral contraceptive use, in vitro fertilization-embryo transfer (IVF-ET), cirrhosis and portal hypertension, and those caused by heritable or acquired factors, including deficiencies of protein C, protein S or antithrombin III [2–4]. Antiphospholipid syndrome (APS) is a systemic autoimmune disease characterized by thrombosis-related clinical disorders or pregnancy morbidity in the presence of antiphospholipid antibodies [5]. APS has been reported to be associated with an increased risk for MVT [6, 7].

Pregnancy itself is an acquired hypercoagulable state. Occurrence of MVT in pregnancy remains reportable with few cases described in the literature. We here describe a pregnant patient who presented with an acute abdomen that was ultimately diagnosed with acute MVT after exploratory laparotomy.

## Case presentation

A 26-year-old primigravid woman, at 35 weeks gestation attended our obstetric department complaining of abdominal pain along with nausea and vomiting for 3 h. The woman’s antenatal care was uneventful. She had no significant medical, surgical or family history and no history suggestive of thromboembolism. She had never used oral contraceptives or any other hormonal therapy.

Upon arrival, she had a temperature of 36.8 °C, pulse rate of 80 beats per minute, respiratory rate of 20 breaths per minute and blood pressure of 119/71 mmHg. A physical examination on admission showed a gravid uterus just below the xiphoid process. No abdominal tenderness or rebound tenderness were appreciated. The bowel sounds were normal and there were no signs suggestive of peritonitis. A hematologic examination revealed a leukocyte count of 13.1 × 10^9^/L (normal range 4.0 × 10^9^/L - 10.0 × 10^9^/L) with neutrophils accounting for 73.9% (normal range 50–70%), hemoglobin level of 98.6 g/L (normal range 100 g/L - 150 g/L), hematocrit of 0.317 (normal range 0.37–0.43), and platelet count of 187 × 10^9^/L (normal range 100 × 10^9^/L - 300 × 10^9^/L). Coagulation profile and biological tests were within normal limits. Obstetric ultrasound revealed a normal fetus compatible with expected gestational age. The fetal monitor showed that fetal heart rate fluctuated between 150 and 160 beats per min and the uterus contracted occasionally. Threatened preterm labor was initially suspected and magnesium sulfate was given to inhibit uterine contractions. Acute gastritis was also considered.

The second day after admission, the patient complained of more intense abdominal pain that was centered in the right lower quadrant, and she experienced increased vomiting and abdominal distention. Physical examination demonstrated right lower quadrant tenderness without rebound tenderness and a distended abdomen and weak bowel sounds. Hematologic testing revealed leukocytosis with a left shift (leucocyte count of 26.9 × 10^9^/L and neutrophils accounting for 91%) and hemoconcentration (hematocrit of 0.439). Serum amylase was normal. An abdominal ultrasound scan revealed dilation and effusion of the right intestinal canals, thickening of the intestinal wall and a small amount of ascites. Abdominal plain radiography was performed and showed no dilated bowel loops and no features of obstruction or pneumoperitoneum. Obstetric ultrasound revealed demise of the fetus.

**Fig. 1:**
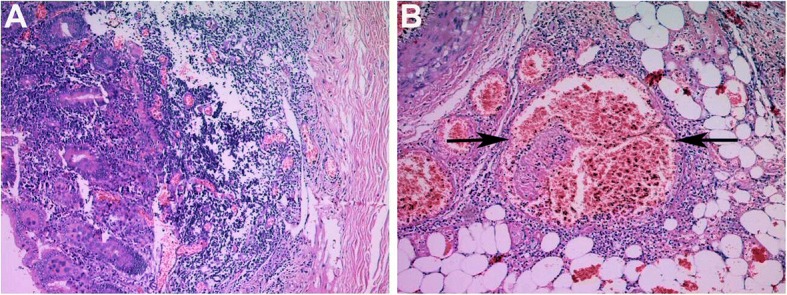
Histopathological images of infarcted intestine and thrombosed mesenteric vein. (**a**) Presence of extensive mucosal denudation and widespread edema, hemorrhage and neutrophil infiltration in the submucosa and muscularis propria of the bowel. (**b**) Presence of thrombosed mesenteric vein (arrow). The sections were stained with hematoxylin and eosin (magnification: × 50)

Acute appendicitis with perforation was suspected and an emergency exploratory laparotomy was performed immediately. On exploration, approximately 1500 mL of serosanguineous peritoneal fluid was found in the abdomen. The entire ileum, part of the jejunum and part of the ascending colon were gangrenous, and thromboembolism in the corresponding mesenteric veins was noted. The necrotic intestine, measuring approximately 180 cm, was resected and an end-to-end jejunum-colon anastomosis was performed. Since there was no evidence of pending spontaneous labor and delivery and to avoid the release of fetally-derived necrotic materials that could complicate the clinical scenario via pro-coagulant and pro-inflammatory effects, a cesarean section was performed and the fetus and placenta were removed. The patient’s hematological values during the operation showed a white cell count of 14.58 × 10^9^/L, neutrophils accounting for 91%, hemoglobin of 73 g/L, a hematocrit of 0.236 and a platelet count of 135 × 10^9^/L. The pathologic examination revealed extensive mucosal denudation, edema, hemorrhage and neutrophil infiltration in the submucosa and muscularis propria of the bowel (Fig. 1a), and widespread thrombosis in mesenteric venous lumens (Fig. 1b).

The patient was transferred to the intensive care unit after surgery. Total parenteral nutrition, intravenous antibiotics and full anticoagulation with low molecular weight heparin were initiated postoperatively and continued until discharge. Thrombolytic therapy was withheld due to bleeding risk. The patient had a reasonably uneventful recovery and was discharged on postoperative day 36. Four years later, the patient was again pregnant and underwent thrombophilia testing (antithrombin, protein C, protein S, and coagulation parameters) at 30 weeks of gestation. At that time, antithrombin III activity was 64% (normal range 80–120%). Her antithrombin III activity returned to normal (96%) 4 weeks later. She delivered a healthy baby through a cesarean section at 38 weeks of gestation without thromboembolic event and without thromboprophylaxis. She has been recurrence-free for 10 years without anticoagulant therapy.

## Discussion and conclusions

MVT is an infrequent condition accounting for 1 in 5000 to 15,000 inpatient admissions, 1 in 1000 emergency department admissions, and 6 to 9% of all cases of acute mesenteric ischemia [8]. MVT is associated with the typical components of Virchow’s triad: hypercoagulability, blood stasis and vascular endothelial injury [8]. Pregnancy is an acquired hypercoagulable state that is associated with an increased risk of thromboembolism [8, 9]. During pregnancy, factors VII, VIIIC, and fibrinogen are increased, while the activity of the fibrinolytic system is decreased [10]. In addition, as pregnancy progresses, the enlarged uterus compresses the inferior vena cava. The resulting increase in blood flow stasis adds to the pro-thrombogenic risk [11].

Still, MVT is very rare during pregnancy. We could find only 14 documented cases of MVT in pregnancy in the English literature after searching MEDLINE using keywords “mesenteric venous thrombosis” and “pregnancy” until June 30, 2018. Of these, 10 cases had precipitating factors in addition to pregnancy and 4 cases were idiopathic. As shown in Table 1, four of these affected pregnant women had a documented inherited thrombophilia, including factor V Leiden gene mutation [12], protein S deficiency [13, 14] and antithrombin deficiency [15]. One woman developed MVT following impregnation via IVF-ET [9], one had mistakenly used oral contraceptive pill and smoked heavily [16, 17], two had surgical emergencies including mid-gut volvulus [18] and a mesenteric cyst [19], one had cytomegalovirus (CMV) infection [20], one had a history of chronic idiopathic MVT [21] and the other four cases had no documented additional risk factors for MVT [10, 11, 22, 23]. Although in our reported case, antithrombin III activity was mildly decreased at a single time point during the patient’s second pregnancy, it returned to normal a month later without any medication. Since the patient had no family history of inherited antithrombin III deficiency and we did not test antithrombin III activity during her first pregnancy, we believe that the occurrence of MVT during her first pregnancy was idiopathic and not secondary to deficiency of antithrombin III (Table 1).

**Table 1:** Incidence of MVT during pregnancy

Case	Ref.	Year	Additional risk	Maternal age	Gestational age (week)	Intestinal resection	Anti-coagulation	Pregnancy outcome
1	Sönmezer, et al. [12]	2004	factor V Leiden gene mutation	32	27	No	Yes	Vaginal delivery at 39 weeks
2	Atakan, et al. [13]	2009	Protein S deficiency	25	20	Yes	Yes	Maternal death
3	Liu, et al. [14]	2014	Protein S deficiency	38	7	Yes	Yes	Vaginal delivery of a normal baby
4	García-Botella, et al. [15]	2016	Antithrombin deficiency	29	7	Yes	Yes	Vaginal delivery at 36 weeks gestation
5	Hirata, et al. [9]	2017	Oral estrogen associated with IVF-ET	34	7	Yes	Yes	Elective abortion
6	Friedman, et al., Graubard and Friedman [16, 17]	1984, 1987	Oral contraceptive	30	14	Yes	Yes	ND
7	Shui, et al. [18]	2011	Mid-gut volvulus	25	35	No	Yes	Cesarean delivery at 35 weeks gestation
8	Giannos, et al. [19]	2017	Mesenteric cyst	27	10	Yes	Yes	Incomplete abortion
9	Zamani, et al. [20]	2009	CMV infection	22	31	Yes	Yes	Fetal death
10	Chan, et al. [21]	2009	Chronic idiopathic MVT	26	7	Yes	Yes	Elective abortion
11	Engelhardt and Kerstein [11]	1989	Idiopathic	32	10	Yes	Yes	Vaginal delivery of a normal baby
12	Foo, et al. [22]	1996	Idiopathic	27	6	No	Yes	Elective abortion
13	Fouad, et al. [10]	2001	Idiopathic	35	28	Yes	Yes	Vaginal delivery at 40 weeks gestation
14	Lin, et al. [23]	2011	Idiopathic	31	34	Yes	Yes	Cesarean delivery at 34 weeks gestation
15	Current report	2018	Idiopathic	26	35	Yes	Yes	Fetal death

CMV, cytomegalovirus; IVF-ET, in vitro fertilization and embryo transfer; MVT, mesenteric venous thrombosis; ND, not described

MVT often becomes part of an ever-worsening pathogenic feedback loop. MVT impedes blood return to the heart, leading to systemic circulatory insufficiency. Thrombosis also causes venous engorgement and bowel ischemia. Rapid and complete occlusion of the mesenteric veins can combine with the inability to expeditiously develop a compensatory collateral circulation to cause transmural bowel infarction. Transmural infarction then promotes the collection of a substantial amount of blood and fluid exudate in the intestinal lumen and peritoneal cavity, thereby fostering hemoconcentration and worsening circulatory insufficiency. Transmural infarction also induces a loss of bowel mucosal integrity, that, in turn allows for bacterial translocation. This can ultimately cause peritonitis, sepsis, multiorgan failure, septic shock, and even death [8].

The hallmark of acute MVT is abdominal pain that is typically described as colicky and midabdominal [2, 8]. Initially, the pain may be mild. In fact, initial physical findings may be entirely normal. With the progression of the disease, the pain can become quite severe but often remains out of proportion to physical signs [2, 8]. Nausea, anorexia, vomiting, and diarrhea are common. Some patients may experience hematemesis, hematochezia, or melena [2, 8]. Abdominal signs such as abdominal tenderness, abdominal distension and ascites may accompany increasing bowel ischemia [2, 8]. Fever, leukocytosis and signs of peritoneal irritation, including rebound tenderness and rigidity, develop later and indicate transmural infarction, bowel gangrene and peritonitis [2, 8]. Dehydration and hemodynamic instability characterized by hypotension, tachycardia and elevated hematocrit are very late manifestations, resulting from the collection of fluid in the bowel lumen and the abdominal cavity [2, 8].

The symptoms of MVT are often remarkably insidious and non-specific. Patients who present with abdominal pain may be mistakenly thought to have gastritis or peptic ulcer, and those with diarrhea may be assumed to have enteritis. When the abdominal pain becomes severe, pancreatitis is often suspected. The early symptoms of MVT in a pregnant woman are often wrongfully interpreted as normal changes of pregnancy [24] or more common pregnancy complications such as spontaneous abortion, preterm delivery, placental abruption, or uterine rupture [19]. Laboratory findings such as leukocytosis and elevated hematocrit are not particularly helpful in winnowing the differential diagnosis [4].

Abdominal plain films show non-specific findings in 50 to 70% of MVT; these include dilated bowel loops, ileus, and thumbprinting from mucosal edema [8]. Contrast enhanced computed tomography (CT) is the diagnostic modality of choice [4, 8]. The diagnostic radiologic finding in MVT is the presence of a venous thrombus manifested as a filling defect in the mesenteric vein. The accuracy of CT is about 90% for the diagnosis of MVT [8]. Other findings suggesting intestinal infarction include bowel wall thickening of greater than 3 mm, thickened mesentery, and indistinct bowel margins [8]. Ascites may also be noted [8]. Homogeneous bowel enhancement along with bowel wall thickening of 10 mm or more has an approximately 90% accuracy for identification of transmural infarction [8]. Bedside Doppler ultrasound can be used to detect large venous thrombi, but this diagnostic modality is operator-dependent and not as sensitive as CT [4]. Magnetic resonance (MR) imaging is another viable testing option, but it offers no distinctive advantage over CT [21]. In addition, the use of MR is limited by expense [8]. Other choices include scintiangiography and angiography, although these are limited by their low sensitivity, restricted availability and invasive nature [4, 8].

Early diagnosis of MVT is hindered by non-diagnostic symptoms, signs and laboratory results, and limited physician awareness of this rare disease. It is reported that only one-third of patients with acute mesenteric ischemia are correctly diagnosed before surgical exploration or death [25]. In the present case, the patient presented with typical abdominal pain, accompanied by nausea and vomiting. Her pain worsened the second day after admission and was out of proportion to the abdominal findings, which demonstrated abdominal tenderness but no abdominal distension, rebound tenderness or rigidity. The documentation of leukocytosis and elevated hematocrit offered no specific clues to the diagnosis of MVT. Preterm delivery, gastritis, and appendicitis rather than MVT were in the initial differential diagnosis. Initial radiologic testing with an abdominal plain film did not suggest bowel infarction. Even though the abdominal ultrasound scan revealed intestinal dilation, thickening of the bowel wall and a small amount of ascites, the treating physicians did not evaluate mesenteric venous flow or the possibility of MVT due to their unfamiliarity with this rare disease. A CT scan was not performed secondary to concerns about fetal risks; the estimated fetal exposure for a typical abdominal radiography is 2.5 mGy, but that of an abdominal CT scan is 30 mGy [26]. Still, diagnostic radiography during pregnancy is not associated with any significant fetal adverse events [27] and necessary testing should be performed when indicated.

Management for MVT includes anticoagulation, surgery and supportive treatment. Anticoagulation with low molecular weight heparin should be initiated as soon as the diagnosis is made, including when diagnosis is delayed until surgery [4]. Although most researchers recommend maintaining anticoagulation therapy for at least 6 months after diagnosis in order to prevent the recurrence of the thrombosis, its benefit is still questioned in some reports [21]. If symptoms worsen or signs of peritonitis or perforation develop during initial management with anticoagulation alone, surgical intervention is warranted. Surgical resection of necrotic bowel and anastomosis are standard. Additional supportive care includes fluid supplementation to correct volume and electrolyte imbalances that result from fluid movement into the bowel lumen and abdominal cavity. Broad spectrum antibiotics are advocated to treat transmural infarction, septic thrombophlebitis, peritonitis, and/or sepsis secondary to intestinal bacterial translocation [4]. Bowel rest and nasogastric aspiration may be needed for patients with abdominal distension, ileus, and severe nausea or vomiting [4, 8].

MVT is extremely dangerous. It is characterized by an insidious onset and early non-specific clinical presentation but can progress very rapidly. Patients with MVT may deteriorate and die after a remarkably short time course due to sepsis, multiorgan failure and shock. In our case, fetal demise within 24 h of the patient’s admission could have been secondary to intrauterine inflammation, compromised uterine blood supply and/or systemic sepsis. Early diagnosis of MVT and timely cesarean section may have allowed for a more favorable fetal outcome.

In summary, MVT in pregnancy is a rare, yet important cause of intestinal ischemia. Non-characteristic symptoms, signs and laboratory results lead to incorrect or late diagnosis of MVT and high maternal and fetal morbidity and mortality. When a pregnant patient presents with an acute abdomen, especially when the symptoms are out of proportion to the signs, MVT should be included in the differential diagnosis. Contrast enhanced CT is a viable choice for the diagnosis of MVT. Anticoagulation therapy should be initiated as soon as the diagnosis is made and surgical intervention should be performed when transmural infarction or peritonitis is suspected.

## Abbreviations

APS: Antiphospholipid syndrome
CMV: cytomegalovirus
CT: computed tomography
IVF-ET: in vitro fertilization-embryo transfer
MR: Magnetic resonance
MVT: Mesenteric venous thrombosis
ND: not described
